# Transient Interference Excision and Spectrum Reconstruction with Partial Samples Using Modified Alternating Direction Method of Multipliers-Net for the Over-the-Horizon Radar

**DOI:** 10.3390/s24092770

**Published:** 2024-04-26

**Authors:** Zhang Man, Quan Huang, Jia Duan

**Affiliations:** 1 School of Electronic and Communication Engineering, Guangzhou University, Guangzhou 510006, China; manzhang401@gzhu.edu.cn; 2 Key Laboratory of On-Chip Communication and Sensor Chip of Guangdong Higher Education Institutes, Guangzhou 510006, China; 3 School of Electronics and Communication Engineering, Sun Yat-sen University, Shenzhen 518107, China; bifiduan119@126.com

**Keywords:** OTHR, modified Alternating Direction Method of Multipliers (ADMM)-Net, spectrum reconstruction, transient interference

## Abstract

Transient interference often submerges the actual targets when employing over-the-horizon radar (OTHR) to detect targets. In addition, modern OTHR needs to carry out multi-target detection from sea to air, resulting in the sparse sampling of echo data. The sparse OTHR signal will raise serious grating lobes using conventional methods and thus degrade target detection performance. This article proposes a modified Alternating Direction Method of Multipliers (ADMM)-Net to reconstruct the target and clutter spectrum of sparse OTHR signals so that target detection can be performed normally. Firstly, transient interferences are identified based on the sparse basis representation and then excised. Therefore, the processed signal can be seen as a sparse OTHR signal. By solving the Doppler sparsity-constrained optimization with the trained network, the complete Doppler spectrum is reconstructed effectively for target detection. Compared with traditional sparse solution methods, the presented approach can balance the efficiency and accuracy of OTHR signal spectrum reconstruction. Both simulation and real-measured OTHR data proved the proposed approach’s performance.

## 1. Introduction

The over-the-horizon radar (OTHR) transmits high-frequency (HF) band (3–30 MHz) signals, and radio signals propagated to the ionosphere are reflected to the ground to detect targets. When compared to typical radar systems, it has advantages such as a great observing range, broad coverage, and anti-concealed capacity [1,2,3,4,5,6]. Even though modern OTHR systems have many benefits, they can have limitations. The OTHR signal is typically contaminated by environmental noise and transient interferences such as air noise, meteor trail echoes, and other electromagnetic devices. Transient interference will submerge potential targets because of its high energy and temporal transience. Generally speaking, the transient interference can raise the noise level by up to 20 dB, observably lowering the target detection ability. Furthermore, modern radar systems must be multi-mode and multi-functional to track and detect several targets simultaneously. In this sense, the time domain signal can be seen as a partly sampled signal. These sparse OTHR signals will generate an exceedingly severe broadening of the clutter during coherent accumulation. As a result, heightened grating lobes in the Doppler spectrum make determining targets challenging.

Three types of techniques are known for eliminating transient interferences that survive the beamforming stage: (1) conventional removal approaches with interference location and subsequent interpolation-based reconstruction; (2) subspace decomposition interpolation-free methods; (3) simultaneous location and removal. The first method is currently widely utilized, which detects and locates interference in the time domain; the typical algorithms corresponding to the first technique can be referred to in [7,8]. The foundations of the first type of technique are comparable: they determine interferences and utilize sequential interpolation to rebuild lost signals. Following clutter filtering, one noticeable strategy is threshold detection based on the high interference-to-noise ratio (INR) technique [7,8,9,10]. However, when clutter and interference overlap, the performance of these methods may degrade. As a result, a matrix-pencil strategy-based interference alleviation method is suggested, which characterizes the interference by its characteristic spectrum dissimilarity. A covariance matrix incorporating noncontaminated samples is developed [11] based on the heightened correlation between clutter and target echoes. A Teager–Kaiser energy operator has been demonstrated to help locate transient interference [12]. After extracting the degraded samples, classic strategies employ interpolation to retrieve misplaced data. An approach to improvement known as weighted forward and backward improvement is presented in [13]. Furthermore, rather than selecting the contaminated samples to zero, a smoothing strategy is introduced to preserve most clutter components. The second category of approaches deteriorates the signal into subspaces and determines interference based on its short-term and high-energy elements. The typical algorithms corresponding to the second type of technique can be referred to in [14]. In [14], an adaptive Gaussian base illustration approach is presented. Following decomposition, the transient interference is directly located based on its local properties. Since the approach requires no interpolation, its actual effect is not affected by the length of coherent integration time. As artificial intelligence approaches advance, several researchers attempt to detect and revamp interference simultaneously. The typical algorithms corresponding to the third type of technique can be referred to [15,16,17], which does not need to detect the location of interferences. On the one hand, matrix decomposition algorithms distinguish transitory interferences [15,16,17]. In [15], the low-rank Hankel matrix recovery technique is introduced to suppress transient interference. Furthermore, [17] presents the Hankel matrix’s sparse and low-rank decomposition. However, choosing the optimal values (e.g., regularization parameters) for these approaches in practical applications is not trivial. On the other hand, deep learning strategies are researched in [18] for transient interference mitigation. An a priori-guided deep learning approach for a frequency-modulated continuous wave radar is developed by treating the interference mitigation problem as a regression problem. In addition, complex empirical mode decomposition [19] and space–time cascaded processing [20] are also used to suppress transient interference. In [19], a transient interference suppression approach based on temporal inverse windowing and the complex empirical mode decomposition technique in the time domain is presented. The method is also useful when the amplitude and the bandwidth of the transient interference are altered. In [20], a space–time two-dimensional cascaded processing approach is presented for transient interference suppression. The approach suggested in [20] can be utilized for transient interference excision while the duration of the transient interference is a long-time coherent processing interval (CPI) or short-time CPI. The performance of this approach is not affected by the duration of transient interference. In general, the imperfections of most existing approaches can be separated into two categories. (1) The calculation is large for simultaneous interference location and removal. Threshold detection approaches cannot be developed for conventional separate processing. (2) The majority of existing approaches ignore the sparse signal circumstances. In these cases, the discontinuous signal will seriously broaden the clutter, ultimately degrading detection and reconstruction.

This article uses an integrated target and clutter spectrum recovery algorithm to perfect target detection for sparsely sampled OTHR signals of modern OTHR systems. A modified Alternating Direction Method of Multipliers (ADMM)-Net based on [21,22] for transient interference excision and spectrum reconstruction for sparse OTHR echoes is presented to address these issues. An adaptive basis is built to describe the returned signal based on the characteristics of transient interferences. After decomposing the produced echo into a sparse basis, signal reconstruction is carried out by filtering the most likely transient interference elements. The ADMM [23,24,25] algorithm is a suboptimal optimization tool for sparse representation problems [26,27]. However, iteration results in a considerable computational cost. The selection of hyper-parameters has a notable impact on solution preciseness. In this article, we expand each iteration of the classic ADMM method into a layer of modified ADMM-Net. Adaptively, the hyper-parameters are learned from OTHR data. In this manner, the reconstruction can be simplified as a forward calculation. Through the enhanced ADMM-Net, it is feasible to identify and extract transient interference simultaneously. This method is unique in two ways: (1) A novel ADMM-Net-based deep learning approach with excellent efficiency and precision is developed by adjusting the transient interference removal problem into a sparse representation problem. (2) Because of the sparse basis decomposition, the ADMM-Net can simultaneously detect interference, remove interference, and rebuild the OTHR signal.

The remainder of the article is arranged as follows: Section 2 describes the sparse signal model for the OTHR. Section 3 provides the modified ADMM-Net for transient interference excision and spectrum reconstruction and provides a detailed explanation of its construction. Section 4 involves the experiments using both simulated and real OTHR data to verify the proposal’s effectiveness.

## 2. OTHR Signal Model

The time-domain signal the OTHR receives for a certain range cell is denoted as $f\left(t\right)$. In general, the received echo can be represented as an additive mixture of ocean-ground clutter echo $c\left(t\right)$, prospective target return $r\left(t\right)$, transient interference $i\left(t\right)$, and other additive interference and noise $n\left(t\right)$. As a result, the signal is defined as the following:

(1) $$f\left(t\right)=c\left(t\right)+r\left(t\right)+i\left(t\right)+n\left(t\right)$$

OTHR illuminates targets from the ionosphere and acquires a considerable amplitude backscattering echo from the geography and sea, known as the clutter signal. Typically, the spectrum of a clutter signal is concentrated within a few Doppler bins. The target signal has a Doppler frequency corresponding to the target’s radial speed. The target signal can be represented as the following:

(2) $$r\left(t\right)=\sum_{n=1}^{N}A_{n}\cdot e^{j2\pi f_{n}t}$$

where N means the number of moving targets in the range gate, $A_{n}$ represents the scattering amplitude, and $f_{n}$ is Doppler frequency of the nth moving target. Due to the radial motion between targets and radar Line Of Sight (LOS), the target echoes raise a Doppler shift. When the Doppler shift is significant adequately, the target signal spectrum is divided from the clutter spectrum, resulting in an increased likelihood of target identification. Nevertheless, the existence of transient interference will overwhelm the target signal.

Transient interference occurs only during a few recurrence periods, but its power is frequently enormous enough to hide the target signal in the Doppler domain, making detection challenging. Transient interference is usually in terms of short-duration features, which can be represented as the following:

(3) $$i\left(t\right)=\sum_{k=1}^{K}A_{k}\cdot rect\left(\frac{t-t_{k}}{T_{k}}\right)\cdot e^{j2\pi f_{k}t}$$

Assume K transient interference signals exist. In Equation (3), $T_{k}$ means the time of the kth transient interference. $t_{k}$ and $f_{k}$ represent the locations in both the time and frequency domains, which denote the corresponding values on the time and frequency axes; $A_{k}$ is the complex amplitude.

**Figure 1:**
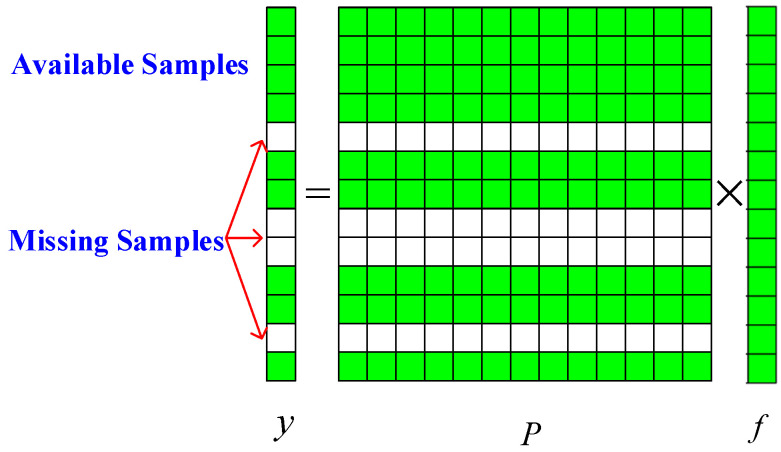
Signal missing diagram.

Assume the OTHR observes ocean and ground targets simultaneously, allocating one of four pulses to ground targets and three to ocean targets. Thus, intermittent sampling occurs. The consequential sparse ocean signal is presented in Figure 1. Green samples are available, but white samples are not because the OTHR switches LOS to view ground targets. Therefore, the white boxes correspond to zero values, and the green boxes correspond to non-zero values.

The discontinuous signal can be represented as the following:

(4) $$y\left(t^{\prime }\right)=Pf\left(t\right)$$

where P means the observed downsampling matrix.

## 3. Modified ADMM-Net for Transient Interference Excision

### 3.1. RWB-SINC Model

Before detecting a target, traditional transient interference excision approaches filter away transient interference in the time domain. While transient interference is removed from the actual signal, it unavoidably results in an absent signal. Typical transient interference excision approaches damage the integrity of the OTHR spectrum and then restore it using interpolation. When the acquired samples are insufficient, error reconstruction is unavoidable. A novel transient interference excision approach is presented to avoid such a procedure. The OTHR signal is decomposed into a collection of width-changeable rectangle window basis–sinc (RWB-SINC). The related parameters of the basis are utilized to determine transient interferences in others. For non-sparse y(t), it can be decomposed into the following form:

(5) $$y\left(t^{\prime }\right)=Pf\left(t\right)=\sum_{p=1}^{\infty }B_{p}\phi_{p}\left(t\right)+n\left(t\right)$$

where $B_{p}$ means the weight of the pth basis; $\phi_{p}\left(t\right)$ represents the spare rectangle window base (RWB); and $n\left(t\right)$ denotes the noise. Since the radar system has restricted bandwidth and beam width, RWB-SINC is appropriate to represent transient interference, target signals, and other clutter signals. The sparse RWB-SINC can be written as the following:

(6) $$\begin{array}{l} \phi_{p}\left(t\right)=rect\left(\frac{t-t_{g}}{T_{g}}\right)\cdot e^{j2\pi f_{g}t}\\ F\left\{\phi_{p}\left(t\right)\right\}=\mathrm{sinc}\left[T_{g}\left(f-f_{g}\right)\right]e^{-j2\pi ft_{g}} \end{array}$$

where the sinc function can be written as $\mathrm{sinc}\left(x\right)=\frac{\sin\left(\pi x\right)}{\pi x}$, and the base changes with parameter set $\Theta_{g}=\left\{T_{g},t_{g},f_{g}\right\}$. $T_{g}$ denotes the time of the signal. $t_{g}$ and $f_{g}$ represent the locations in both the time and frequency domains, which denote the corresponding values on the time and frequency axes. Obviously, this basis $\phi_{p}\left(t\right)$ is an RWB function in the time domain and SINC-like in the frequency domain. The parameters help the base adjust to approximate the local behaviors of decomposed signals, allowing signals in the time or frequency domains to be represented precisely. Subsequently, the weight $B_{p}$ and time width $T_{g}$ can be utilized to recognize transient interference.

After utilizing the RWB model to characterize the original OTHR signal, a power threshold is utilized to recognize transient interference from the evaluated components with a temporary period. According to [7], it is indicated that a power threshold located in the scope from ${\left(1.3{\left\|B\phi_{small\_deviation}\right\|}_{\min}\right)}^{2}$ to ${\left(2.5{\left\|B\phi_{small\_deviation}\right\|}_{\min}\right)}^{2}$ is the most appropriate empirical where ${\left({\left\|B\phi_{small\_deviation}\right\|}_{\min}\right)}^{2}$ means the lowest power in the miniature variation elements. After utilizing the RWB representative to describe the original signal, a power threshold is employed to recognize transient interference from the removed components briefly.

### 3.2. Modified ADMM-Net for Spectrum Reconstruction

**Figure 2:**
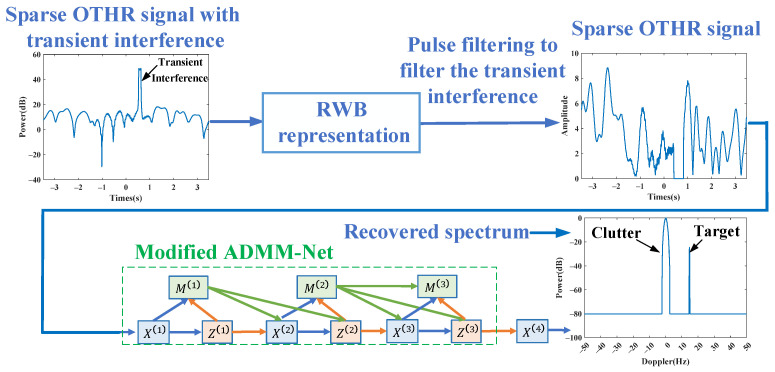
The transient interference excision and spectrum reconstruction process for a sparse OTHR signal utilizing a modified ADMM-Net.

After transient interference excision utilizing the RWB-SINC model, the compressed sensing method can reconstruct the spectrum from limited sampled OTHR signals. In this article, a modified ADMM-Net is used to reconstruct the spectrum of the sparsely sampled OTHR signals. The whole procedure is illustrated in Figure 2. Subsequently, we will present the modified ADMM-Net.

The spectrum reestablishment case can be identical to a sparse retrieval issue, such as the following:

(7) $$\hat{x}=\underset{x}{\mathrm{argmin}}{\left\|y-\Phi Px\right\|}_{2}^{2}+\sum_{k}\lambda_{k}g\left(\phi_{k}x\right)$$

where y denotes the sparsely sampled OTHR signals, x represents the original spectrum, ΦP is the corresponding downsampling matrix, $\lambda_{k}$ is the regularization parameter, and $g\left(\cdot \right)$ means the regularization function. The parameter group $\Theta_{g}=\left\{T_{g},t_{g},f_{g}\right\}$ of Equation (6) can be illustrated from the related dictionary $\phi_{k}$.

The above optimization issue can be handled efficiently by employing the ADMM algorithm. By presenting additional variables $z=\left\{z_{1},z_{2},\cdots ,z_{K}\right\}$, Equation (7) is identical to the following:

(8) $$\underset{x,z}{min}\frac{1}{2}{\left\|y-\Phi Px\right\|}_{2}^{2}+\sum_{k}\lambda_{k}g\left(z_{k}\right)\;\;\;\;\;s.t.\;\;\;\;\;z_{k}=\phi_{k}x$$

Its augmented Lagrange function can be rewritten as the following:

(9) $$\begin{array}{ll} \zeta_{p}\left(x,z,\alpha \right)= & \frac{1}{2}{\left\|y-\Phi Px\right\|}_{2}^{2}+\sum_{k=1}^{K}\lambda_{k}g\left(z_{k}\right)\\ & -\sum_{k=1}^{K}\langle \alpha_{k},z_{k}-\phi_{k}x\rangle +\sum_{k=1}^{K}\frac{\rho_{k}}{2}{\left\|z_{k}-\phi_{k}x\right\|}_{2}^{2} \end{array}$$

where $\alpha =\left\{\alpha_{k}\right\}$ denotes the Lagrangian multiplier, $\rho =\left\{\rho_{k}\right\}$ represents penalty parameters, and $\beta_{k}=\alpha_{k}/\rho_{k}$. The ADMM approach handled three subproblems alternatively as follows:

(10) $$\left\{\begin{array}{l} X^{\left(n\right)}:x^{\left(n\right)}=F^{H}{\left(P^{T}P+\sum_{k=1}^{K}\rho_{k}F\phi_{k}^{H}\phi_{k}F^{H}\right)}^{-1}\\ \;\;\;\;\;\;\;\;\;\;\;\;\;\;\;\;\;\;\;\;\;\cdot \left[P^{T}y+\sum_{k=1}^{K}\rho_{k}F\phi_{k}^{H}\left(z_{k}^{\left(n-1\right)}-\beta_{k}^{\left(n-1\right)}\right)\right],\\ Z^{\left(n\right)}:z_{k}^{\left(n\right)}=S\left(\phi_{k}x^{\left(n\right)}+\beta_{k}^{\left(n-1\right)};\lambda_{k}/\rho_{k}\right),\\ M^{\left(n\right)}:\beta_{k}^{\left(n\right)}=\beta_{k}^{\left(n-1\right)}+\eta_{k}\left(\phi_{k}x^{\left(n\right)}-z_{k}^{\left(n\right)}\right). \end{array}\right.$$

where $x^{\left(n\right)}$ can be usefully processed by fast Fourier transform (FFT); $S\left(\cdot \right)$ denotes a nonlinear function; and $\eta_{k}$ represents an update speed.

However, the classic ADMM approach requires a lot of work to select the nonlinear function for the general regularization function. Moreover, adjusting the hyper-parameters is not effortless. A modified deep ADMM-Net is developed to unfold the iterative processes to an information discharge diagram to address such difficulties. After this, the network learns the nonlinear function and optimizes parameters from the OTHR data, enhancing accuracy in signal reestablishment. With the learned optimized parameters, the testing process forwards the modified ADMM-Net without iterations and is computationally efficient.

Since we model the RWB-SINC extraction into a reestablished instance, the modified ADMM-Net can be adjusted quickly and accurately to handle such an issue. We created a fixed sampling matrix for the ADMM-Net by setting $T_{g}=\Delta T\cdot m,t_{g}=n\Delta t,f_{g}=k\Delta f$.

**Figure 3:**
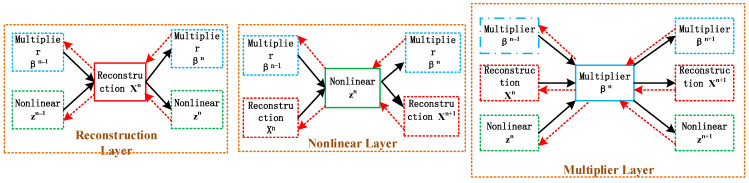
The structure of the modified ADMM-Net.

The modified ADMM-Net is represented in Figure 3. In the structure, each step in Equation (8) is described by a partition with three stages. The Lagrange multiplier updating mechanism expressed in Equation (10) means the multiplier update layer. The signal reestablish layer reconstructs the OTHR signal utilizing the procedure described in Equation (10). The nonlinear layer reaches nonlinear modifications employing the piecewise linear function $S\left(\cdot \right)$, which is set equality from −1 to 1. The piecewise linear function $S\left(\cdot \right)$ is expressed as follows:

(11) $$S\left(a;{\left\{p_{i},q_{k,i}^{\left(n\right)}\right\}}_{i=1}^{N_{c}}\right)=\left\{\begin{array}{c} a+q_{k,1}^{\left(n\right)}-p_{1},\;\;\;\;\;\;\;\;\;\;\;\;\;\;\;a<p_{1}\\ a+q_{k,N_{c}}^{\left(n\right)}-p_{N_{c}},\;\;\;\;\;\;\;\;\;\;\;\;\;\;\;a>p_{N_{c}}\\ q_{k,i}^{\left(n\right)}+\frac{\left(a-p_{k}\right)\left(q_{k,i+1}^{\left(n\right)}-q_{k,i}^{\left(n\right)}\right)}{p_{k+1}-p_{k}},p_{1}\leq a\leq p_{N_{c}} \end{array}\right.$$

where $i=\left\lfloor \frac{a-p_{1}}{p_{2}-p_{1}}\right\rfloor ,{\left\{p_{i}\right\}}_{i=1}^{N_{c}}$ is set equality from −1 to 1, and ${\left\{q_{k,i}\right\}}_{i=1}^{N_{c}}$ are the values related to the k-th filter in the nth stage. Note that normalization should be carried out for this module.

The modified ADMM-Net is designed by piling several partitions jointly, which learn the subsequent optimized parameters in a network structure: $\rho_{k}$ in the phase of signal regeneration, $q_{k,i}$ in the phase of the nonlinear module, and $\eta_{k}$ in the phase of multiplier update. The dictionaries are constructed ahead of time, and there are K filters. In Equation (6), the signal resolution is characterized as nonlinear channel Z with known regularization. The optimized parameters are recovered from the signal utilizing an amplitude threshold to the nonlinear maximum weight allocations.

The thoroughly sampled OTHR signal spectrum is utilized as the label signal $x^{gt}$, while the partially sampled data y are employed as an input of the ADMM-Net. The training set is created from numerous pairings of partially sampled OTHR data and original OTHR signals. Normalized mean square error (NMSE) is selected as the loss function, which can be represented as the following:

(12) $$E\left(\Theta \right)=\frac{1}{X_{set}}\sum_{\left(y_{t},x_{t}^{dt}\right)}\frac{\sqrt{{\left\|\hat{x}\left(y,\Theta \right)-x^{gt}\right\|}_{2}^{2}}}{\sqrt{{\left\|x^{gt}\right\|}_{2}^{2}}}$$

where training set $X_{set}$ is designed to include pairs of under-sampled OTHR signals and the original spectrum. The optimized parameters are learned by minimizing Equation (12) employing a golden section search (GSS) algorithm. The parameter $\rho_{k}$ was originally set as 0.02, $\eta_{k}=0.6$, and 51 points have a uniform value of 0.1 for the nonlinear function. These parameters represent the initialization of network parameters, which will change based on initial parameters in the network training process.

Back-propagation (BP) updates the optimized parameters using the modified ADMM-Net. The ADMM-Net is trained through BP, as shown by the dotted line in Figure 3. The gradients of each phase consist of the inputs and phase parameters. The value of the network’s data must be complex to rebuild a signal from the complex OTHR data. The network’s parameters are real values. Consequently, the gradients can be calculated as the following:

(13) $$\begin{array}{l} \frac{\partial E}{\partial I}=\mathrm{real}\left(\frac{\partial E}{\partial O}\right)\mathrm{real}\left(\frac{\partial O}{\partial I}\right)+j\cdot \mathrm{imag}\left(\frac{\partial E}{\partial O}\right)\mathrm{imag}\left(\frac{\partial O}{\partial I}\right)\\ \frac{\partial E}{\partial \Theta }=\mathrm{real}\left(\frac{\partial E}{\partial O}\right)\cdot \mathrm{real}\left(\frac{\partial O}{\partial \Theta }\right)+\mathrm{imag}\left(\frac{\partial E}{\partial O}\right)\cdot \mathrm{imag}\left(\frac{\partial O}{\partial \Theta }\right) \end{array}$$

where I,O, and Θ are the input, output, and optimized parameters of the layer. $\mathrm{real}\left(\cdot \right)$ and $\mathrm{imag}\left(\cdot \right)$ represent the real and imaginary parts of a function, respectively. Since the OTHR signal has complex values, the value of the network’s data must be complex in order to rebuild a signal. On the other hand, the parameters learned (such as step size, Lagrange multiplier, etc.) from the modified ADMM-Net are real values. So, Equation (13) has two forms when calculating the gradient. The first term corresponds to the loss function to calculate the partial derivative of the complex data, and the second term corresponds to the loss function to calculate the partial derivative of the real parameter.

After training the modified ADMM-Net, rerunning the forward process with the learned optimized parameters can calculate the OTHR signal rebuild and signal decomposition. Moreover, changing the input matrix permits the ADMM-Net to perform with different sampling matrices.

## 4. Experimental Results and Analysis

This section displays experimental results to illustrate our proposed method’s transient interference excision and spectrum reconstructive capability.

### 4.1. Simulated Experiments

**Figure 4:**
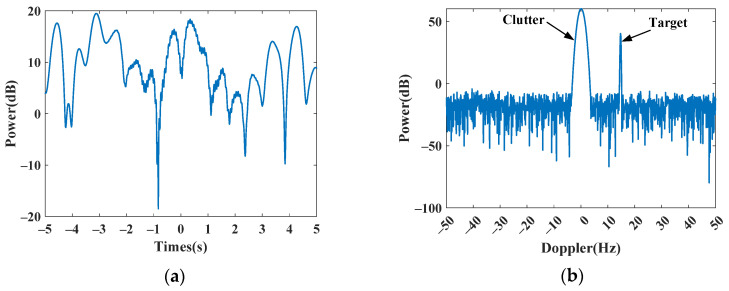
Simulated signal without transient interference. (**a**) Time-domain signal. (**b**) Doppler spectrum.

**Figure 5:**
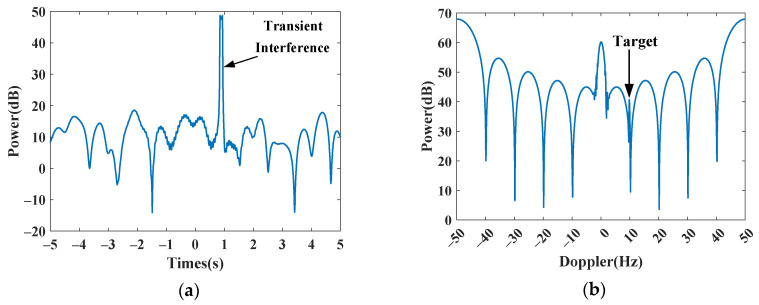
Simulated signal with transient interference. (**a**) Time-domain signal. (**b**) Doppler spectrum.

**Figure 6:**
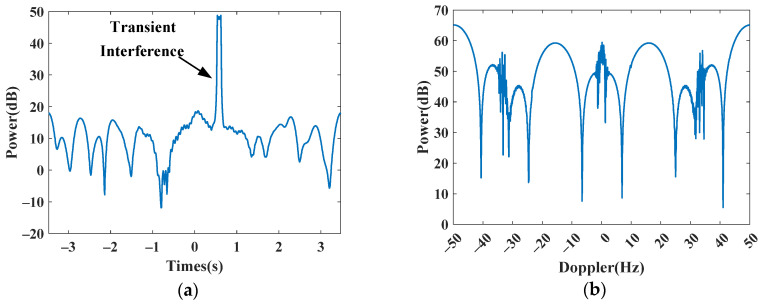
Simulated signal with transient interference after subsampling. (**a**) Time-domain signal. (**b**) Doppler spectrum.

In this subsection, we first use simulated data to validate the Doppler spectrum reconstruction method for sparse OTHR signals with transient interferences. For clarity, the simulated parameters are listed in Table 1. For reference, the time-domain signal and its spectrum without interference are shown in Figure 4a,b. Afterward, an interference is added to the signal to generate a contaminated signal with a signal-to-interference ratio (SIR) of −40 dB. The signal-to-clutter ratio (SCR) is −20 dB. As shown in Figure 5, the target is submerged by the interference in the spectrum domain, resulting in the dramatic performance degradation of target detection. The received signal is sparsely sampled because of the multi-function working mode of the OTHR. Here, we generate discontinuously sampled OTHR signals by regularly removing one from four pulses. The time and spectrum of sparse OTHR signals are displayed in Figure 6. The discontinuous sampling further degrades the SIR. It is hard to discriminate the target from the clutter, making target detection impossible.

To remove the transient interference and recover the signal spectrum simultaneously, our proposed modified ADMM-Net is conducted to validate its effectiveness.

**Figure 7:**
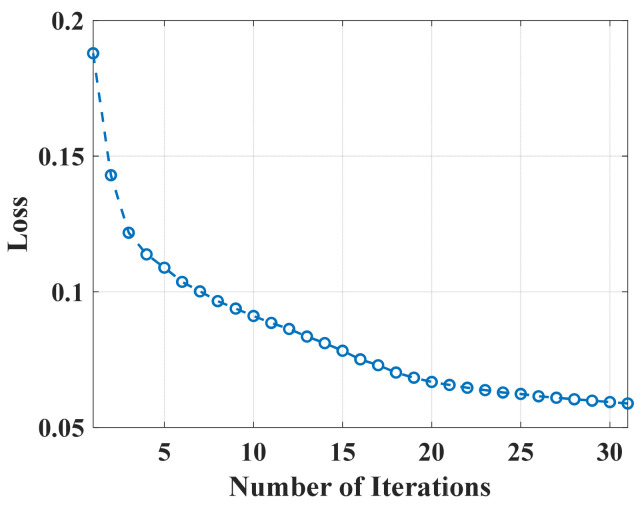
Change in loss function in the process of network training.

**Figure 8:**
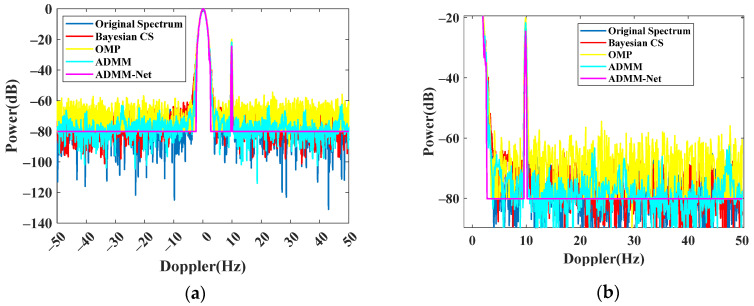
Recovered spectrum comparison. (**a**) Recovered spectrum. (**b**) Zoomed spectrum.

First, the ADMM-Net is trained on the constructed training data set. This training set includes several OTHR simulation signals with transient interference. Due to the lack of an open, sparsely sampled OTHR data set, we generate corresponding data sets at sparse sampling rates (the sampling rate represents the length of the effective signal in the total signal) of 80%, 75%, and 66.66%, respectively. Each sparse rate includes 30 sets of spectrum data and subsampled OTHR signals with transient interference, 25 groups used for training and 5 for testing. In the network training process, the loss function change is shown in Figure 7. After 30 epochs, the training loss converges to 0.059. Then, the reconstructed signal can be generated by replacing the subsampling matrix P, as shown in Figure 8. For comparison, the results of Bayesian CS [28,29], orthogonal matching pursuit (OMP) [30], and the traditional ADMM algorithm (30 iterations) [25] are also shown. The proposal can recover the spectrum precisely and rapidly. By transforming the iteration with traditional recovery, the reconstruction is accomplished by forwarding the operation with automatically learned parameters, which can promise a high and precise signal recovery. Compared with other methods, it is superior in precision and time efficiency.

### 4.2. Comparison Experiments

Several metrics are defined to provide precise results for a quantitative comparison of the proposal with available methods. The similarity of the reconstructed signal and the reference are evaluated by the normalized root mean square error (NRMSE). The signal-to-clutter ratio (SCR) is also computed to describe the performance in subsequent detection. The assessment of the time consumption of each signal is carried out to evaluate their efficiency.

In this subsection, to assess the effect of the sparsity on proposals, different sampling ratio cases are generated for comparison. The corresponding network must be trained according to the sparse rate for different sparse sampled data. Here, network training is carried out at sampling rates of 80%, 75%, and 66.66%. The signal-to-clutter ratio (SCR) of the original spectrum is −20 dB. The results are listed in Table 2. Although the OMP can reach a high precision, the time consumption is beyond acceptance. Compared with other traditional iteration methods except OMP, the proposed modified ADMM-Net can achieve the most efficient time consumption while having high reconstruction capability. The forward operation of the modified ADMM-Net is only equal to six iterations of the traditional ADMM approach. Thus, the calculation speed has been improved.

**Figure 9:**
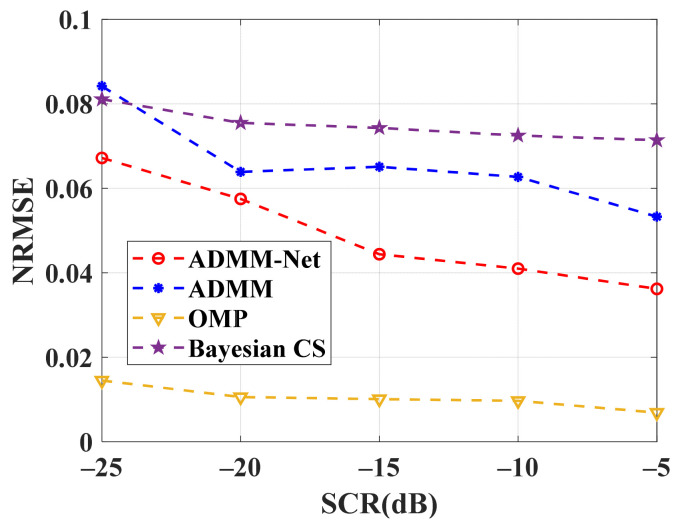
NRMSE comparison of modified ADMM-Net and traditional CS reconstruction approaches versus SCR.

Experiments with different SCRs were carried out to study further the influence of SCR on the proposed method’s spectrum recovery. Figure 9 shows the results. The modified ADMM-Net can achieve better OTHR signal spectrum recovery faster than the traditional method. Although OMP has the best effect, the calculation required for the OMP method is too large and unsuitable for practical application.

### 4.3. Real Data Experiments

In this subsection, we testify to the performance of CS-based spectrum reconstruction with partially measured data. The measured data were obtained with an over-the-horizon radar (OTHR) in China on 11 December 2009; detailed information on the system can be found in [6]. The measured data were processed using the previously trained network, while the sparse sampling rate was identical. In our experiment, the data used contained 1024 echo cells.

**Figure 10:**
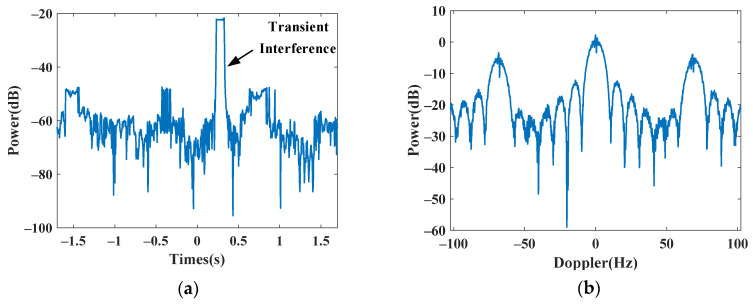
Subsampled data from real OTHR data. (**a**) Time-domain signal. (**b**) Doppler spectrum.

Only one range-gate data point is used to simulate discontinuous samples from multimode operation. We generate discontinuous sampled signals by removing one from four pulses regularly. Due to the missing partial sample, serious grating lobes appear in the Doppler spectrum, as shown in Figure 10b, which prevents successful target detection in the Doppler domain. Its time-domain envelope and Doppler spectrum are shown in Figure 10a and Figure 10b, respectively.

**Figure 11:**
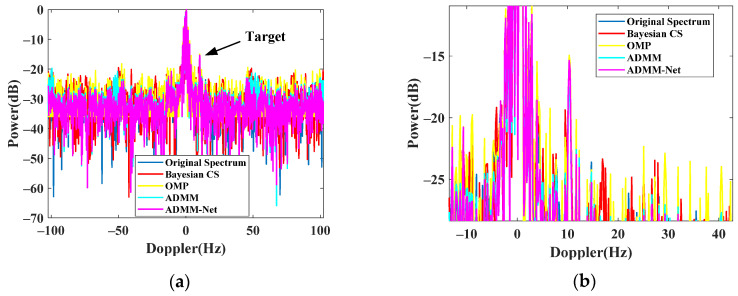
Complete spectrum after transient interference excision. (**a**) Recovered spectrum. (**b**) Zoomed spectrum.

By applying the trained ADMM-Net-based spectrum reconstruction method to the discontinuous signal, we can recover the full Doppler spectrum with high quality. In Figure 11, we compare the proposal and traditional methods in the Doppler domain. The proposal can achieve the perfect recovery of a complete signal without missing a quarter of the whole samples. Compared with other methods, the proposal can achieve the highest SCR (−15.36 dB) for perfect target detection.

## 5. Conclusions

This article presents a modified ADMM-Net to reconstruct the target and clutter spectrum from the sparse OTHR signal. First, the transient interferences are identified and excised based on the sparse basis representation. The modified ADMM-Net is produced by unrolling the iterations of ADMM into a data flow map. The trained network can solve the spectrum reconstruction issue of the sparse OTHR signal. Thus, the complete Doppler spectrum is obtained, which can be used for target detection. The proposed approach can balance the efficiency and accuracy of OTHR signal spectrum reconstruction. Both simulation and real data experiments validate the effectiveness.

## Figures and Tables

**Table 1:** Simulated parameters.

Parameter	Value	Parameter	Value
Clutter Doppler bandwidth	6 Hz	PRF	100 HZ
Doppler shift of target	9.7656 Hz	Azimuth Cell	1024
Signal-to-clutter ratio	−20 dB	SNR	10 dB
Signal-to-interference ratio	−40 dB		

**Table 2:** The effect of the sparsity ratio.

Sparsity Ratio	80%		75%		66.66%		Time Consumption (CPU)
Metric	NRMSE	SCR (dB)	NRMSE	SCR (dB)	NRMSE	SCR (dB)	
ADMM-Net	0.0678	−20.96	0.0575	−20.87	0.0652	−21.09	0.319 s
ADMM	0.0783	−20.76	0.0639	−20.46	0.0629	−20.96	0.514 s
Bayesian CS	0.0778	−20.66	0.0755	−20.89	0.0905	−20.95	6.49 s
OMP	0.0167	−20.06	0.0106	−20.03	0.0905	−20.05	12.6 s

## Data Availability

Data underlying the results presented in this paper are not publicly available at this time but may be obtained from the authors upon reasonable request.
